# Melanopsin Retinal Ganglion Cells and Pupil: Clinical Implications for Neuro-Ophthalmology

**DOI:** 10.3389/fneur.2018.01047

**Published:** 2018-12-07

**Authors:** Chiara La Morgia, Valerio Carelli, Michele Carbonelli

**Affiliations:** ^1^Unità Operativa Complessa Clinica Neurologica, IRCCS Istituto delle Scienze Neurologiche di Bologna, Ospedale Bellaria, Bologna, Italy; ^2^Dipartimento di Scienze Biomediche e Neuromotorie, Università di Bologna, Bologna, Italy

**Keywords:** melanopsin retinal ganglion cells, light, pupil, neurodegeneration, optic nerve, optic neuropathies, Alzheimer, Parkinson

## Abstract

Melanopsin retinal ganglion cells (mRGCs) are intrinsically photosensitive RGCs that mediate many relevant non-image forming functions of the eye, including the pupillary light reflex, through the projections to the olivary pretectal nucleus. In particular, the post-illumination pupil response (PIPR), as evaluated by chromatic pupillometry, can be used as a reliable marker of mRGC function *in vivo*. In the last years, pupillometry has become a promising tool to assess mRGC dysfunction in various neurological and neuro-ophthalmological conditions. In this review we will present the most relevant findings of pupillometric studies in glaucoma, hereditary optic neuropathies, ischemic optic neuropathies, idiopathic intracranial hypertension, multiple sclerosis, Parkinson's disease, and mood disorders. The use of PIPR as a marker for mRGC function is also proposed for other neurodegenerative disorders in which circadian dysfunction is documented.

## Introduction

Melanopsin retinal ganglion cells (mRGCs) are intrinsically photosensitive RGCs expressing the photopigment melanopsin (1, 2). They constitute about 0.2–1% of total RGCs and contribute to the photoentrainment of circadian rhythms, through their projections to the suprachiasmatic nucleus (SCN), but also to other anatomical structures devoted to non-image forming functions of the eye. These include pupil regulation through their projections to the olivary pretectal nucleus (OPN) in the midbrain (3–5) and brain structures relevant for emotional processing (6). Recent data support the notion that distinct subpopulations of mRGCs mediate different functions in the central nervous system, including circadian rhythm regulation and pupil light reflex (PLR) through their projections to the OPN (5, 7).

The mRGC contribution to the pupil function has been extensively investigated over the recent years and it is now clear that rods mediate mainly the transient pupil contraction, whereas mRGCs contribute to the steady-state pupil constriction (8–11). In fact, mRGCs are characterized by a unique property, which is the capability of firing without fatigue in response to continuous stimulation, consistent with the intrinsic activation of these cells (12). In particular, post-illumination pupil response (PIPR), measured after 1.7 s from onset of the light stimulus, and its magnitude can be considered as specific measures of mRGC function (13). Different protocols, using different light paradigms and experimental setting of stimulation, have been tested and now established to assess *in vivo* PLR mediated by mRGCs (13, 14). Specifically, the contribution of mRGCs to pupil response has been evaluated using blue (470 nm) and red (640 nm) light, being the blue light able to maximally stimulating mRGCs (13–15).

The PLR mediated by mRGCs has been investigated in different ophthalmological conditions including glaucoma (16–18), retinitis pigmentosa (19), diabetes (20), Leber's congenital amaurosis (14), age-related macular degeneration (21), and ischemic optic neuropathies (22, 23). Moreover, various neurological and psychiatric disorders have been evaluated, including hereditary optic neuropathies (24–29), seasonal affective disorder (SAD) (30), idiopathic intracranial hypertension (IIH) (31, 32), multiple sclerosis (MS) (33), and Parkinson's disease (PD) (34).

In this review we will focus on pupillometry findings in neuro-ophthalmological disorders in which pupil and circadian functions have been investigated. In particular, we include disorders affecting the optic nerve such as glaucoma and hereditary optic neuropathies, neurodegenerative disorders with optic nerve involvement and circadian dysfunction and affective disorders for which a relevant role of mRGCs has been postulated. We will highlight the potential role of mRGC-mediated pupil function as an *in vivo* objective tool and possible biomarker for evaluating mRGC function in different neurodegenerative disorders.

## Melanopsin RGCs and pupil in glaucoma and Anterior Ischemic Optic Neuropathy

Glaucoma is a chronic optic neuropathy characterized by loss of peripheral visual field secondary to a progressive and extensive loss of RGCs and their optic nerve fibers (35). The pathophysiology of glaucoma is not yet completely understood, even though two common and pivotal events are the increase in intraocular pressure and impaired microcirculation (vascular deregulation), both preceding the RGC death (36). Previous studies in monkey models of glaucoma reported that all classes of RGCs are susceptible to injury or damage since the early stages of the disease including the sub-population of mRGCs (37). Concordantly, recent clinical studies have shown high prevalence of sleep and circadian disorders, as well as depression in glaucoma patients, implying that the mRGC-driven photoentrainment of circadian rhythms may be affected in patients with glaucoma (38–41).

In the last years, several studies were published aimed at measuring *in vivo* the integrity of mRGC system in glaucoma by assessing the PLR (16–18). Overall, the results and the conclusions of these studies have been frequently inconsistent because of the different protocols and methodology adopted for chromatic pupillography. In fact, many variables may affect the results, such as time of dark adaptation, light stimulus (duration, intensity, and wavelength), time to measure the intrinsic melanopsin-mediated PIPR, direct or consensual pupil stimulation, and so on (see Table 1).

**Table 1:** Pupillometry findings in glaucoma and in anterior ischemic optic neuropathy.

	**Population**	**PLR Methods**	**Main findings**
Kankipati et al. (17)	16 glaucoma patients 19 healthy controls	10 s light stimulus of blue (470 nm) or red (623 nm) to one eye after dilation (60°). Consensual PIPR: average pupil diameter over a period of 30 s, starting 10 s after light offset minus baseline pupil diameter	Patients net PIPR (blue PIPR minus red PIPR) was significantly smaller than in controls and inversely correlated with the MD in visual field of the tested eye
Feigl et al. (16)	25 glaucoma patients 16 healthy controls	10 s blue (488 nm) and red (610 nm) stimuli presented to the right eye, and the consensual pupil response of the left eye was measured (7°) PIPR: average pupil diameter 20–50 s after light offset	The blue PIPR was significantly smaller between controls and patients with advanced glaucoma, as well as between early and advanced glaucoma patients
Nissen et al. (18)	11 unilateral glaucoma patients 11 healthy controls	10 s of darkness (baseline pupil), 20 s of exposure stimulus (red-660 nm and blue-470 nm) and 50 s of darkness (post-exposure). The area under the curve (AUC) of consensual pupil was calculated for: (1) during the 20 s of light-on, (2) during the first 10 s after light was turned off and (3) from 10 to 30 s after light was turned off (AUC30–50 s)	The pupillary response to blue light was decreased in the glaucomatous eyes of unilateral glaucoma. In the unaffected eyes, the pupillary response to blue light did not differ from that of healthy controls
Rukmini et al. (42)	40 glaucoma patients 161 healthy controls	Narrowband blue (469 nm) or red (631 nm) (After 1 min dark adaptation). Pupillary constriction amplitude (%) after 2-min irradiance of gradually increasing light stimuli (ranging from 6.8 to 13.8 Log photons/cm^2^/s)	In glaucomatous eyes, reduced pupillary responses to high-irradiance blue light were associated with greater visual field loss (MD) and optic disc cupping
Kelbsch et al. (43)	25 glaucoma patients 16 Ocular Hypertension (OH) patients 16 healthy controls	28 lx, red (605 nm) or blue (420 nm) light with a duration of either 1 or 4 s. The consensual PIPR was recorded	PIPR blue-red was reduced in glaucoma patients compared to normals (*p* < 0.001) and OH (*p* < 0.01). There was no significant difference between OH and controls. PIPR was inversely correlated with MD in the tested eye
Münch et al. (27)	11 LHON patients 11 glaucoma patients 22 healthy controls	Post-stimulus pupil size at 6 s from light offset (1 s stimulus red and blue) was recorded before, and immediately after light exposure (2 h of bright light exposure)	Only glaucoma patients demonstrated a relative attenuation PRL and at advanced stages of disease also melatonin suppression abnormal response
Adhikari et al. (44)	12 glaucoma suspects 22 early glaucoma patients 12 late glaucoma patients 21 healthy controls	(After 10 min dark adaptation) Post-stimulus pupil size at 6 s from light offset (1 s, blue-464 nm, 15.5 log quanta.cm^−2^ s^−1^ blue light presented in the supero-nasal quadrant field)	Supero-nasal field melanopsin PIPR measurements differentiated mRGC dysfunction in glaucoma suspects and early glaucoma from healthy controls and showed a linear correlation with RNFL thickness
Najjar et al. (45)	46 early stage glaucoma patients 90 controls	Pupillary constriction amplitude (%) after 2-min irradiance of gradually increasing light stimuli (ranging from 8.5 to 14.5 Log photons/cm^2^/s) for blue light (462 nm) and (from 8.5 to 14 Log photons/cm^2^/s) for red light (638 nm)	Maximum amplitude of pupil constriction was reduced in patients with early-stage glaucoma compared with controls for blue and red stimuli. This reduction was dependent on the irradiance of the light exposure, and showed a linear correlation with RNFL thickness
Herbst et al. (23)	10 unilateral NAION patients 11 controls	Consensual pupil responses during and after exposure to continuous 20 s blue (470 nm) or red (660 nm) light of high intensity (300 cd/m^2^) were recorded in each eye	Compared with the responses of the controls, the blue light post-illumination pupil responses were similar in the affected eyes and increased in the fellow non-affected eyes. This suggests a possible adaptive phenomenon, of ipRGCs in both eyes
Tsika et al. (22)	10 unilateral NAION patients 8 bilateral AION patients (1 NAION and 7 AION associated with optic disc drusen) 29 controls	Post-stimulus pupil size (PSPS) at 6 s following monocular as well as binocular light stimulation of 1 s (red-635 nm, blue-464 nm) at different intensities (1.0, 1.5, and 2.0 log cd/m^2^)	PSPS to all monocularly-presented light stimuli were impaired in AION eyes. To binocular light stimulation, the PSPS of AION patients was similar to controls

Nonetheless, it is now clearly proven that the PLR, and particularly the PIPR, is altered in moderate and advanced stages of glaucoma, despite the use of different chromatic illumination paradigms (16–18, 27, 42, 43). These findings are also correlated with functional and structural features of the glaucomatous pathology, as demonstrated by the fact that PIPR is inversely correlated with the mean deviation in the visual fields (17, 42, 43). Moreover, an inverse correlation between PLR to high-irradiance blue light and optic disc cupping measured by Heidelberg Retinal Tomography was found (42), and the reduction of PLR to blue and red light correlates with retinal nerve fiber layer (RNFL) thinning (44, 45). These results are in line with a study demonstrating that there is a correlation between the severity of the glaucoma and the reduction of the PIPR (16). This is concordant with the knowledge that in glaucoma the central 10 degrees of the retina, where the mRGCs are more concentrated, are affected only in the last stages of the disease. However, in the last 2 years, a new method of light delivery (quadrant field pupillometry) (44), and a new light stimulation protocol (increasing light regimens) (45) were used to better investigate the pre-perimetric and early-stage glaucoma. By stimulating only the portion of the retina most precociously affected in glaucoma it was shown that the superonasal quadrant PIPR differentiates patients suspected of having glaucoma and with early glaucoma from healthy controls, and this finding correlated with RNFL thinning measured by OCT (44). Furthermore, by increasing logarithmically the light stimuli intensity, PLR is reduced in patients with early-stage glaucoma compared with controls at moderate to high irradiances with both blue and red light, and the maximal pupillary constriction amplitude is correlated to the RNFL thickness (45). To highlight the possible correlation of different measurements of mRGC functions, it is also worth mentioning that in advanced glaucoma, individuals with greater light-induced melatonin suppression (a measure of the retino-hypothalamic tract function) have also a smaller PIPR (27).

Finally, a functional damage of the mRGC-mediated PLR has been reported in the affected eyes of patients with unilateral or bilateral anterior ischemic optic neuropathy (AION), specifically 10 patients with unilateral non-arteritic ischemic optic neuropathy (NAION), 1 bilateral NAION, and 7 patients with bilateral AION associated with optic disc drusen, compared to the unaffected and control eyes (22). Differently, previous studies failed to demonstrate differences in the PLR between NAION and control eyes (23). Furthermore, if the bright blue stimuli were presented bilaterally and simultaneously to both eyes, bilateral AION patients showed, through binocular summation, the same post-stimulus pupil size of patients with unilateral AION and controls (22).

## Melanopsin RGCs and Pupil in Hereditary Mitochondrial Optic Neuropathies

Mitochondrial optic neuropathies are inherited disorders of the optic nerve due to mitochondrial DNA (mtDNA) mutations affecting the mitochondrial-encoded subunits of complex I of the respiratory chain complex, pathogenic for Leber's hereditary Optic Neuropathy (LHON) or to mutations of the nuclear gene *OPA1* causing Dominant Optic Atrophy (DOA) (46, 47). These inherited mitochondrial disorders are characterized by the selective loss of RGCs, in particular those originating the papillo-macular bundle, thus leading to optic atrophy secondary to mitochondrial dysfunctions with the invariable outcome of severe visual loss (46). In both disorders, previous data suggested the maintenance of the PLR even in the chronic stage of the disease, pointing to a pupil-visual dissociation (48, 49). In fact, in these disorders recent histological studies demonstrated a relative preservation of mRGCs compared to the massive loss of regular RGCs in both LHON and DOA, which supports the maintenance of the PLR in these patients (50). At this regard, interestingly, a previous post mortem study demonstrated the relative sparing of the retinofugal fibers to the pretectum in a LHON case, supporting the maintenance of the mRGC projections to the pretectum, which constitute the afferent pathway of the PLR (51). The reasons for the robustness of mRGCs in mitochondrial optic neuropathies are still unknown and under investigation, even though the possible role of peculiar metabolic properties, including the size of the soma, has been proposed (50, 52, 53). More recently, pupillometric studies showed a relative maintenance of the mRGC-mediated pupil response in LHON and DOA patients (24–29) (Table 2). Similarly to the maintenance of the PLR a preserved light-induced melatonin suppression has been demonstrated in LHON and DOA patients supporting a relative preservation of these cells in hereditary mitochondrial optic neuropathies (50). Interestingly, the preservation of mRGCs and PLR has also been demonstrated in an OPA1-mouse model (54).

**Table 2:** Pupillometry findings in neurological disorders.

	**Population**	**PLR Methods**	**Main findings**
Moura et al. (24)	10 LHON patients 16 controls	1 s red (640 nm) and blue (470 nm) light flashes at 1, 10, 100, and 250 cd/m2 luminance Monocular undilated stimulation, patch of the other eye	Overall maintenance of PLR in LHON patients despite the severity of optic atrophy
Kawasaki et al. (25)	1 LHON patient (14448/ND6) one eye affected	20 s red (660 nm) and blue (470 nm) light at 100 and 300 cd/m^2^ in affected and unaffected eye	Similar sustained PLR in the affected and unaffected eye suggesting preservation of mRGCs in the affected eye
Kawasaki et al. (26)	8 HON patients 8 controls	1 or 30 s red (635 ± 20 nm) (1 cd/m^2^) and blue (463 ± 26 nm) (−4 to 2.5 log cd/m^2^) light Simultaneous stimulation of both undilated eyes	No significant difference between HON and controls in terms of PLR parameters
Münch et al. (27)	11 HON patients 11 glaucoma 22 controls	1 s or 30 s light stimulus at 635 ± 20 nm (red light) and 464 ± 26 nm (blue light) Simultaneous stimulation of both undilated eyes	Similar sustained response after blue light in HON patients compared to controls
Nissen et al. (28)	29 OPA1 mutation patients carrying the c.983A > G (*n* = 14) or c.2708_ 2711delTTAG mutation (*n* = 15) 40 controls	Isoluminant (300 cd/m^2^) red (660 nm) or blue (470 nm) light flash (20 s) Monocular stimulation and recording of the controlateral eye	No differences between OPA1 patients and controls in terms of PIPR
Loo et al. (29)	5 OPA1 patients 54 controls	Red (631 nm) and blue (469 nm) light stimulation (order of light exposure random) gradually increasing intensity from 6.8 to 13.8 log photons/cm^2^/s1 over 2 min (preceded and followed by 1 min of darkness)	Dose-response curve (mean constriction amplitude) for blue and red light similar between OPA1 patients and controls
Roecklein et al. (30)	15 SAD patients 15 controls	Red (632.9 nm) and blue (467.7 nm) 30 s light stimuli presented to both eyes and pupil recorded in LE	Reduced PIPR and lower PIPR percent change to blue light in SAD compared to controls
Park et al. (31)	13 IIH patients 13 controls	1 s blue and light flashes (rod, melanopsin and rod conditions) Monocular undilated stimulation, patch of the other eye	Smaller PLRs (transient and sustaineide response) under melanopsin and rod paradigms in IIH patients compared to controls
Ba-Ali et al. (32)	13 drug-naïve IIH patients 13 controls		No difference in melanopsin-driven PLR
Meltzer et al. (33)	24 MS patients 15 controls	1 s red (622 nm) and blue (463 nm) administered alternatively to each eye (max 2.6 log lux)	Reduced PIPR (melanopsin-driven PLR) to blue light in MS eyes with thinner GCL + IPL and with previous optic neuritis
Joyce et al. (34)	17 PD patients 12 controls	Pulsed (8 s) or phasic (12 s) blue (465 nm) or red (638 nm) light stimulation Recording of the consensual response with the stimulated eye dilated	Reduced PIPR and pupil constriction amplitude in PD patients compared to controls

## Melanopsin RGCs and Pupil in Other Neurological Disorders

In the last years the mRGC-mediated pupil light response has been investigated in various neurological disorders, including IIH, MS, and PD (31–34).

In a cohort of 13 IIH patients compared to 13 controls it was reported a significant reduction of PLR under melanopsin and rod paradigms in IIH subjects, suggesting the potential use of these parameters as an objective measure of RGC dysfunction in IIH (31). However, the abnormal mRGC-driven PLR has not been reported in a cohort of drug naïve IIH patients (32).

A significant reduction of the sustained pupil response to blue light in the eyes with thinner ganglion cell layer (GCL) + inner plexiform layer (IPL) was demonstrated in a group of 24 MS patients, in particular in those with a previous history of optic neuritis, compared to 15 controls (33). The authors proposed the use of the sustained pupil response to light mediated by mRGCs as a surrogate biomarker for neurodegeneration, including the retinohypothalamic tract, in MS patients (33). In consideration that mRGCs are a fundamental conduit for circadian photoentrainment, the sustained PLR to light may be used as a surrogate marker for RHT integrity and consequently for circadian measurements including melatonin rhythm. This may be relevant for potential light therapeutic interventions in these patients (33). Congruently, previous studies demonstrated an abnormal melatonin rhythm in MS patients (55).

An attenuated PIPR for short wavelength and reduced pupil constriction amplitude for long wavelength stimulation was described in a group of 17 early PD patients compared to a control group (34). Pupil metrics in this group were not influenced by disease severity, sleep quality, medications, or OCT measurements and were controlled for unrest pupil conditions. The authors proposed the pupil response mediated by mRGCs as potential biomarker for non-motor symptoms in PD, such as sleep and circadian dysfunction (34). In fact, there is a large body of evidence supporting the occurrence of circadian dysfunction in PD (56).

Finally, a recent study reported the occurrence of PLR dysfunction in R6/2 and Q175 Huntington's disease (HD) mouse models, with a prevalent contribution of cone dysfunction in young-middle-aged mice and of mRGCs in old mice (57). HD is a neurodegenerative disorder in which circadian dysfunction is a prominent and early disease trait pointing again to a possible mRGC dysfunction (56, 58).

Based on these recent findings, it seems reasonable that other neurological disorders, for which there is evidence of circadian dysfunction and mRGC pathology, such as Alzheimer's disease (AD) (59, 60), HD (56–58), and possibly others, may present an abnormal mRGC-driven PLR.

## Melanopsin RGCs and Pupil in Affective Disorders

SAD is a psychiatric condition characterized by the recurrence of depression in winter, in relation to low levels of ambient light in this season (61). Even if the etiology of this disorder is still elusive, the possible role of individual seasonal variation in retinal sensitivity, and in particular of retinal subsensitivity in SAD has been proposed (62–64). Moreover, a polymorphism in the melanopsin (*OPN4*) gene (P10L) has been associated with SAD, suggesting that mRGCs and sensitivity to light may play a relevant role in the pathogenesis of SAD (65). Based on these premises, Roecklein and coauthors investigated the PIPR in 15 individuals with SAD compared to 15 controls. They found a reduced PIPR and a lower PIPR percent change in response to blue light in SAD subjects compared to controls, implying an abnormal mRGC-mediated response to light, as measured by PLR in SAD (30). Moreover, the PIPR response after blue light varied in relation to the OPN4 I394T genotype, another polymorphic variant, suggesting again a possible influence of genetic predisposition in modulating the sensitivity to light in SAD (30). Interestingly, this polymorphic variant has also been found to influence the steady-state pupil diameter in controls (66).

Differently, the melanopsin-mediated PIPR measurements were not significantly different between eight patients with non-seasonal depression and 13 age-matched healthy controls matched for day-light exposure (67). This finding possibly implies a different pathophysiological mechanism in SAD and non-seasonal depression. However, another study using a different light stimulation protocol, showed an abnormal PIPR in both seasonal-depressed and non-seasonal depressed patients (68).

## Discussion

Intrinsically photosensitive retinal ganglion cells, the mRGCs, are unique photoreceptors located in the inner retina, which express the photopigment melanopsin (1, 2, 7). The presence of melanopsin makes these cells maximally sensitive to blue light at 470–480 nm and able to spontaneously spike for a long period, even when isolated from the surrounding retinal structures (7, 12). The mRGCs are crucial for non-image forming functions of the eye including circadian photoentrainment, sleep and melatonin synthesis, and PLR. Of particular importance, in this context, is the possibility of using some pupil metrics, such as the PIPR, as a specific signature of mRGC function *in vivo* (8, 13, 56) for ophthalmological and neurodegenerative disorders, which may present circadian dysfunction. In fact, mRGCs contribute mainly to the sustained component of the PLR and, using blue wavelength light, it is possible to isolate the melanopsin contribution to the PLR.

The availability of the mRGC-mediated PLR as a tool to indirectly test the circadian system status, as recently proposed (69), opens new avenues in the analysis of circadian, sleep, and non-motor features in many neurodegenerative disorders. Interestingly, it has been demonstrated in 15 healthy subjects, using combined evaluations including pupillometry, actigraphy, light sensors and body temperature, a close inverse relationship between pupil light response metrics and circadian status (70). In particular, for the pupil recordings it was used a protocol in which the right eye was dilated and different light stimuli including different light wavelength were tested (5 min stimuli) with 40 min of darkness between the light stimulations. Pupil parameters were analyzed using *ad-hoc* software. For the actigraphic recordings the subjects wore an actigraph with light sensor and non-parametric circadian measures, such as intradaily variability, interdaily stability, relative amplitude, L5 and M5, were obtained (70). The authors proposed the Circadian Status Index as an integrative measure to unify three aspects (robustness, timing, and level) of the three circadian rhythm measures (temperature, activity, and light), as well as a global parameter for pupil metrics (circadian photoreception PLR). However, the authors found an inverse relationship between the pupil and circadian metrics. These contradictory findings between circadian status robustness and the PLR might be referred to individual differences in the M1 cell population of mRGCs. Larger studies, more uniform light stimulation protocols and the inclusion of more circadian and pupil metrics are warranted to analyze the possible correlations between pupil metrics and circadian status. In fact, the current available pupillometric studies all suffer the limitation of great heterogeneity of stimulation protocols and consequent lack of reproducibility of their results. Similarly, all these studies are generally underpowered by the limited number of subjects analyzed.

Finally, since mRGCs are contributing to other non-visual functions of the eye, and different class of mRGCs have different projections to the CNS contributing to different functions (5), it must be emphasized that the finding of an abnormal mRGC-mediated PLR does not mean necessarily a global dysfunction of these cells. Overall, the availability of an easily accessible metric for mRGC function, in conjunction with other tests, such as melatonin suppression test, actigraphic recordings, and functional MRI, may represent a comprehensive strategy to further exploring the function of these cells in patients with different neuro-ophthalmological conditions.

## Conclusions and future directions

In conclusion, the use of PLR mediated by mRGCs, as a measure of mRGC function, is of particular relevance for neurodegenerative disorders for which there is already evidence of circadian and sleep dysfunction, such as PD, AD, and HD. Similarly, it might be also relevant for other neurological disorders with evidence of circadian dysfunction such as frontotemporal dementia (71), Lewy-Body dementia (72), Progressive Supranuclear Palsy (73, 74), and possibly prion diseases, in particular fatal familial insomnia (75). Moreover, the study of PLR mediated by mRGCs might be particularly intriguing for conditions, in which light sensitivity is a predominant feature, such as photophobia (76, 77) and photosensitivity in epilepsy (78). At this regard, an abnormal PLR has been recently documented in migraineous photophobic subjects, even though it was not specifically assessed the mRGC contribution to PLR (79, 80).

Overall, after adequate standardization of light protocols, the availability of an easy accessible tool to assess mRGC function, as a surrogate marker for more general non-image forming functions of the eye, including circadian rhythms and sleep, is a particularly promising biomarker for neurodegenerative disorders.

## Author Contributions

CLM and MC were responsible for conception, design, drafting, and revision of the manuscript. VC was responsible for conception and revision of the manuscript.

### Conflict of Interest Statement

The authors declare that the research was conducted in the absence of any commercial or financial relationships that could be construed as a potential conflict of interest.
